# The Accuracy of Molecular Detection Targeting the Mutation C2611T for Detecting Moderate-Level Azithromycin Resistance in *Neisseria gonorrhoeae*: A Systematic Review and Meta-Analysis

**DOI:** 10.3390/antibiotics10091027

**Published:** 2021-08-24

**Authors:** Qian Zhou, Jingwei Liu, Shaochun Chen, Wenqi Xu, Yan Han, Yueping Yin

**Affiliations:** National Center for STD Control, Chinese Center for Disease Control and Prevention, Department of Reference STD Lab, Institute of Dermatology, Chinese Academy of Medical Sciences and Peking Union Medical College, Nanjing 210042, China; zhouqian14@pku.edu.cn (Q.Z.); jingwei.liuchn@gmail.com (J.L.); chensc@ncstdlc.org (S.C.); xuwq@ncstdlc.org (W.X.); hany@ncstdlc.org (Y.H.)

**Keywords:** *Neisseria gonorrhoeae*, 23S rRNA, azithromycin resistance

## Abstract

Background: *Neisseria gonorrhoeae (N. gonorrhoeae)* is now recognized as a commonly reported sexually transmitted pathogen, and the increasing drug resistance of *N. gonorrhoeae* has become a serious public health problem. The accuracy of molecular detection for detecting moderate-level azithromycin resistance is not well-established. We summarized the data from studies of the *N. gonorrhoeae* 23S rRNA mutation at position 2611 with azithromycin resistance to determine the relationship between the mutation and resistance. Methods and Findings: In this systematic review and meta-analysis, two researchers independently searched six databases for studies with data for the azithromycin minimum inhibitory concentrations (MICs) and the 23S rRNA mutation C2611T of each *N. gonorrhoeae* isolate. Since the breakpoint of moderate-level resistance to azithromycin (ML-AzmR) was not determined, we divided the moderate level into two groups according to the range of MICs (moderate resistance limited to 2–128 mg/L or 4–128 mg/L) for data extraction. A random-effects model was used to calculate the pooled sensitivity rate, the specificity rate, the pooled positive likelihood ratio (PLR), the negative likelihood ratio (NLR), and the diagnostic odds ratio (DOR). Meta-regression analyses by detection method, isolates sampling (a random sample or not), location, and sample size were performed to explore the possible causes of heterogeneity. The potential publication bias of the included studies was conducted by the Deeks’ test. We included 20 studies in our study: 20 studies have data of *N. gonorrhoeae* with MICs between 2 and 128 mg/L with mutation or without mutation at position 2611(4759 samples), and 14 studies have data of *N. gonorrhoeae* with MICs between 4 and 128 mg/L (3367 samples). In the group with the moderate level of 2–128 mg/L, the pooled sensitivity rate of the molecular assays was determined to be 71.9% (95% CI, 67.6–74%), the pooled specificity rate was 98.7% (95% CI, 98.2–99.0%), and the DOR ranged from 55.0 to 351.3 (mean, 139.1). In the 4–128 mg/L group, the pooled sensitivity rate was 91.9% (95% CI, 88.9–94.2%), the pooled specificity rate was 95.9% (95% CI, 95.1–96.6%), and the DOR ranged from 41.9 to 364.1 (mean, 123.6). Conclusion: Through this meta-analysis, we found that the C2611T mutation of 23S rRNA is valuable for the molecular diagnostic of moderate-level azithromycin resistance (ML-AzmR) in *N. gonorrhoeae*, especially when the moderate level is set at 4–128 mg/L. This rapid molecular detection method can be used for the rapid identification of ML-AzmR isolates in the clinic.

## 1. Introduction

*Neisseria gonorrhoeae* (gonococcus) is the etiologic agent of gonorrhea, a sexually transmitted infection (STI) that remains a major global public health concern [1]. In China, gonorrhea is the second most commonly reported sexually transmitted disease only after syphilis, and 117,938 new gonorrhea cases were reported in 2019 [2]. The global public health burden due to gonorrhea is also high; the WHO estimated that there were 86.9 million incident global cases of gonorrhea among 15–49-year-old adults, and the global prevalence of gonorrhea was as high as 0.9% in 2016 [3].

In recent years, gonococcus has become progressively resistant to a wide range of antibiotics, include tetracyclines, penicillin, and sulfonamides [4]. As a result of the significant decline in the efficacy of available antimicrobials, *N. gonorrhoeae* has been identified as an emerging public health problem. In 1990, the WHO Global Gonococcal Antimicrobial Surveillance Programme (WHO GASP) was established to monitor gonococcal antimicrobial resistance (AMR) worldwide [5]. Nowadays, a combination therapy of ceftriaxone plus azithromycin is the most widely recommended treatment for gonorrhea in the United States [6] and the United Kingdom [7]. However, there is also an increasing prevalence of *N. gonorrhoeae* strains with resistance to azithromycin [8]. On the basis of the minimum inhibitory concentrations (MICs), *N. gonorrhoeae* isolates can be categorized into high-level azithromycin-resistant (HL-AzmR), moderate-level azithromycin-resistant (ML-AzmR), and low-level azithromycin-resistant (LL-AzmR). The azithromycin MICs of HL-AzmR *N. gonorrhoeae* isolates are commonly defined as ≥256 mg/L. However, it is still controversial whether the lower limit of ML-AzmR *N. gonorrhoeae* isolates’ MICs is 2 or 4 mg/L [9]. According to the laboratory diagnosis guideline of the WHO and EUCAST, the methods currently used for determining MICs include the agar dilution method and the E-test method. In the CLSI guidelines, disk diffusion or agar dilution MIC tests are routine for clinical testing. Disk diffusion only enables qualitative testing of antimicrobial resistance. Of the two methods used for the quantitative determination of MICs, the agar dilution method is complex and time-consuming, and the E-test method is cost-intensive and is not applicable for routine diagnostics [4]. As a new method to detect drug resistance, the molecular detection method is widely used in the detection of penicillinase-producing *N. gonorrhoeae* (PPNG), tetracycline-resistant *N. gonorrhoeae* (TRNG), and chromosomally mediated penicillin-resistant *N. gonorrhoeae* (CMRNG) [10], but not in the routine use of azithromycin-resistant *N. gonorrhoeae*.

The resistance to azithromycin of *N. gonorrhoeae* is primarily associated with 23S rRNA point mutations. The 23S rRNA of *N. gonorrhoeae* is an important target for azithromycin to bind to and exert its toxicological effect by interfering with mRNA. There are four alleles in the operon gene of the V region of 23S rRNA. The mutation sites are C2611T and A2059G (*E. coli* coding system), but C2611T is more common. It is generally thought that the mutation of the 2611 site is related to the moderate level of azithromycin resistance, while the A2059G mutation can lead to high levels of azithromycin resistance [11]. Because of the above phenomena, researchers can determine the intermediate level of azithromycin resistance of by rapid molecular detection methods, such as PCR or WGS technology. Compared with the agar dilution method or the E-test method, these methods are simple and fast.

So far, there is no clear study on the accuracy of 23S rRNA mutation C2611T detection for azithromycin resistance. In this study, we systematically evaluated the sensitivity and specificity of molecular detection targeting the mutation C2611T for detecting middle-level azithromycin resistance in *N. gonorrhoeae*. Through this study, we provide a basis for further research into the rapid and accurate detection of 23S rRNA point mutant *N. gonorrhoeae* using the molecular detection method.

## 2. Methods

This study was registered with PROSPERO, number CRD42021248296.

### 2.1. Literature Search and Study Selection

The present study was carried out following the Preferred Reporting Items for Systematic reviews and Meta-Analyses (PRISMA) guidelines [12]. Two researchers independently searched six databases (PubMed, Embase, Web of Science, China National Knowledge Infrastructure, and Wanfang Database, last search completed in June 2021) using the terms ((((2611) OR (2599) OR (23S)) OR (rRNA)) and (((((azithromycin) OR (Antimicrobial)) OR (azithromycin)) OR (resistan *)) OR (suscep *)) and ((Neisseria gonorrhoeae) OR Neisseria gonorrhoeae) to identify relevant studies. We searched articles published in English and Chinese and reviewed the references of studies to identify other relevant studies. All the references were uploaded into Endnote Software.

Search results were first screened based on the title and abstract, and any studies that appeared to meet the eligibility criteria, or where eligibility was unclear, progressed to full-text screening. Studies included in our meta-analysis had to be consistent with the following criteria: (1) the research was published in English or Chinese; (2) the study indicated the number of moderate-level azithromycin resistance and non-moderate-level azithromycin resistance *N. gonorrhoeae* isolates; (3) the study indicated the results of molecular assays targeting position 2611 of the 23S rRNA gene.

### 2.2. Data Extraction

Using a standardized form, data were extracted from each included article. The extraction process was carried out by two independent reviewers, with referral to a third reviewer if necessary. It is still controversial as to whether the lower limit of the ML-AzmR *N. gonorrhoeae* isolates’ MIC is 2 or 4 mg/L, so we divided them into two groups (the moderate-level drug resistance breakpoint was 2–128 or 4–128 mg/L) to extract the article data for meta-analysis. Information extracted from all studies included: (1) title, first author, publication year, study country, study period; (2) the technique used for detecting the mutation C2611T; (3) isolates selection (was the sampling continuous or random); (4) the numbers of true positives (TPs), false positives (FPs), false negatives (FNs), and true negatives (TNs) (Table 1). Some of the included studies did not report these figures directly in the results section but showed relevant data in the supplementary tables or discussion sections instead. Therefore, we extracted the relevant data from these sections.

### 2.3. Quality Assessment

To assess the methodological quality of the eligible studies, the QUADAS2 tool was used to examine bias in studies of diagnostic accuracy by two independent investigators (Z.Q. and L.J.) [13]. According to the tool, we assessed the risk of bias from the following four aspects: patient selection, the index test, the reference standard, and flow and timing. Individual risk of bias information was combined to provide an assessment of the overall quality of the evidence. Review Manager V.5.3 software was used to generate pictures of the results.

### 2.4. Statistical Analyses

We took the moderate-level azithromycin resistance phenotype of *N. gonorrhoeae* detected by the agar dilution method and the E-test method as the gold standard. The numbers of ML-AzmR *N. gonorrhoeae* isolates with mutation or without mutation, and non-ML-AzmR isolates with mutation or without mutation were defined as true positive (TP), false negative (FN), false positive (FP), and true negative (TN), respectively. Meta-Disc 1.4 [14] and Stata 15.1 (Stata Corp, College Station, TX, USA) software was used for the meta-analysis. The sensitivity rate, specificity rate, pooled positive likelihood ratio (PLR), negative likelihood ratio (NLR), diagnostic OR (DOR) and their corresponding 95% CIs were calculated using a random-effects model. The summary receiver operating characteristic (sROC) curve was plotted, and the area under the sROC curve was calculated to evaluate the overall accuracy of the molecular analysis of the mutant C2611T in identifying ML-AzmR *Neisseria gonorrhoeae* isolates. The heterogeneity was evaluated by performing the Q test and calculating I^2^ values. I^2^ > 50%, or a *p*-value for heterogeneity less than 0.1, indicated high heterogeneity. To find the source of the heterogeneity of the results, the Spearman correlation coefficient and meta-regression analysis were applied. Meta-regression analysis was performed according to the detection method, isolates sampling (was a random sample or not), location, and sample size. The Deeks’ funnel plot asymmetry test was used to detect potential publication bias.

## 3. Results

### 3.1. Study Selection

**Figure 1:**
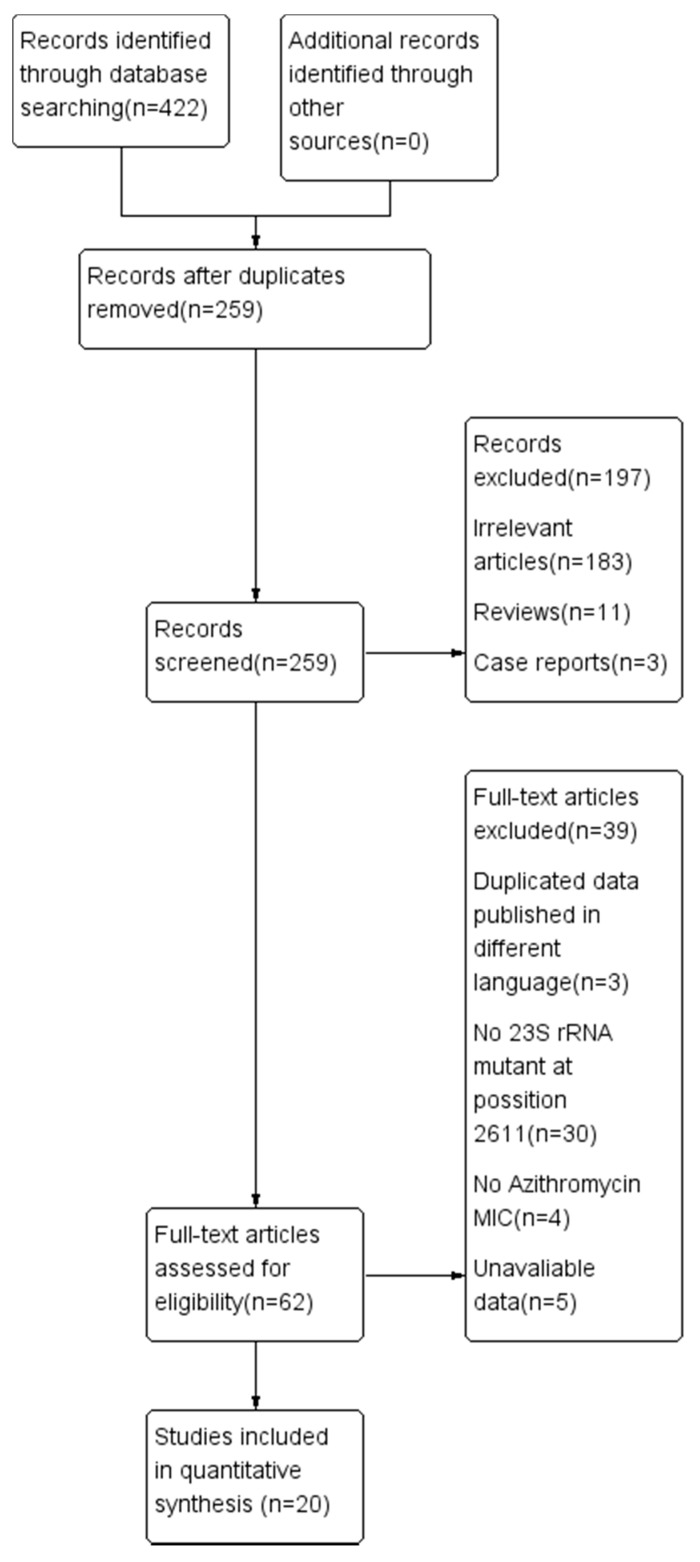
Flow diagram for selecting published studies for meta-analysis according to PRISMA guidelines, generated by Review Manager Software (RevMan version 5.3).

A total of 422 potentially relevant abstracts were identified, of which 259 were duplicates and thus removed. The remaining 259 abstracts were assessed; 197 of them were irrelevant articles, reviews, and case reports and were subsequently excluded. The remaining 62 full-text articles were assessed for eligibility and 42 of them were excluded because they consisted of duplicated data published in a different language, or they did not indicate azithromycin MICs or the 23S rRNA mutant at position 2611. There were 20 studies that met the inclusion criteria for the meta-analysis [15,16,17,18,19,20,21,22,23,24,25,26,27,28,29,30,31,32,33,34] (Figure 1). The data and characteristics of the 20 studies are summarized in Table 2 and Table 3.

### 3.2. Quality Assessment

**Figure 2:**
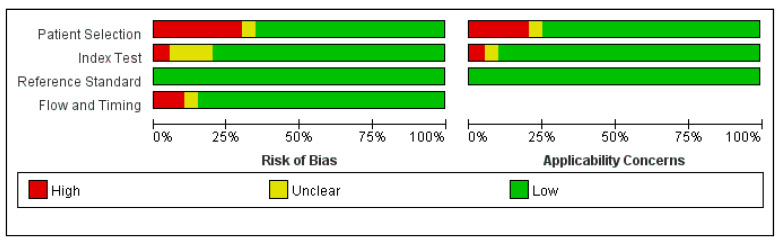
Quality evaluation of the included studies.

**Figure 3:**
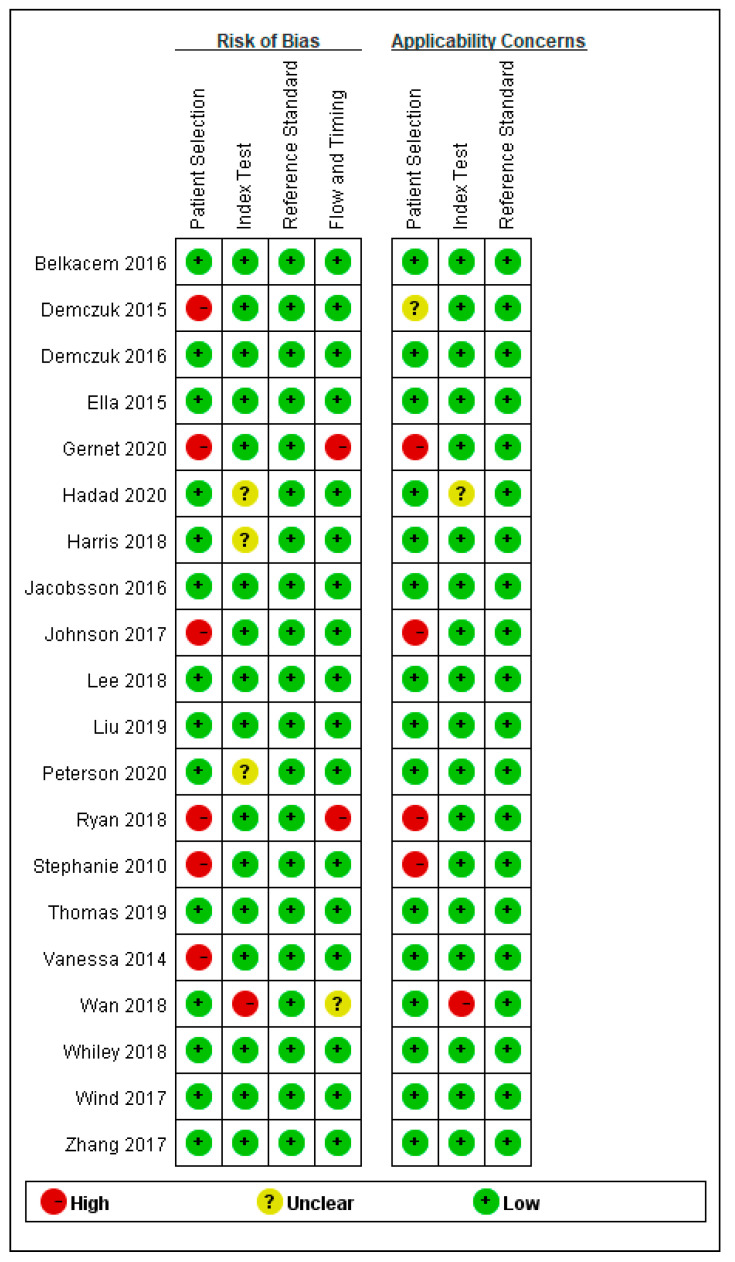
Risk of bias and concerns summary.

Risk of bias results are summarized in Figure 2 and Figure 3. Thirty percent of the studies had a high risk of selection bias because these studies were not continuous or random in the selection of strains. No reference standard bias was found in any of the studies.

### 3.3. Meta-Analysis

#### 3.3.1. Meta-Analysis of the Group with Moderate Resistance Limited to 2–128 mg/L

**Figure 4:**
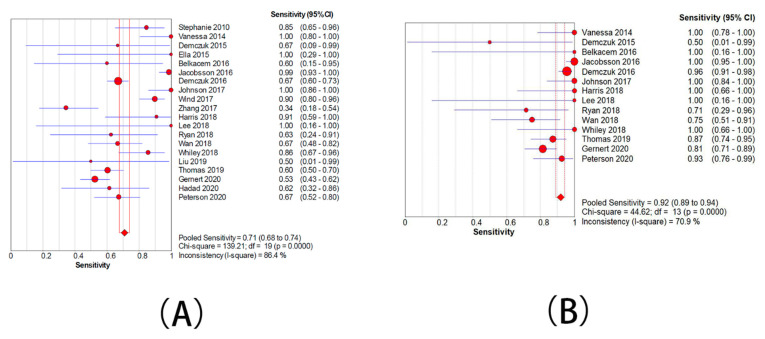
Forest plots for the combined sensitivity from included studies. (**A**) The group with moderate resistance limited to 2–128 mg/L. (**B**) The group with moderate resistance limited to 4–128 mg/L.

**Figure 5:**
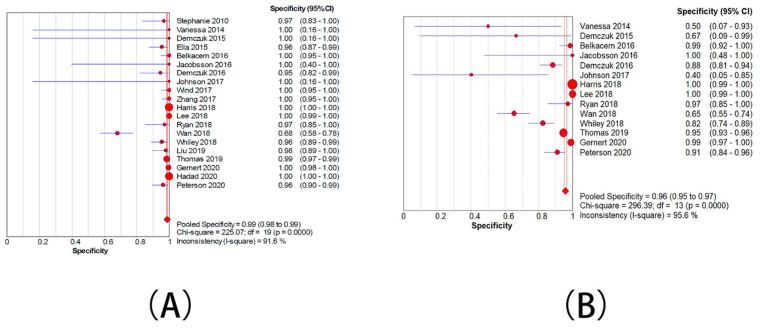
Forest plots for the combined specificity from included studies. (**A**) The group with moderate resistance limited to 2–128 mg/L. (**B**) The group with moderate resistance limited to 4–128 mg/L.

**Figure 6:**
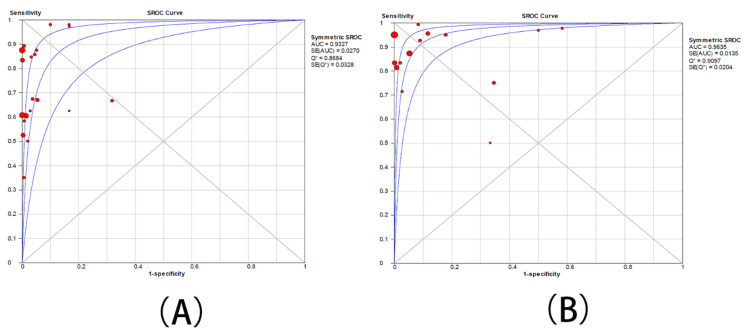
sROC curve of fourteen studies with both sensitivity and specificity rates. (**A**) The group with moderate resistance limited to 2–128 mg/L. (**B**) The group with moderate resistance limited to 4–128 mg/L. Q* defined by the point where sensitivity and specificity are equal.

Twenty studies presented data for determining the sensitivity rate of detection of ML-AzmR *N. gonorrhoeae* isolates (MICs was 2–128 mg/L) based on the mutation C2611T. The sensitivity rates in these studies ranged from 34.5% to 100.0%. The pooled sensitivity rate of the molecular assays was determined to be 71.9% (95% CI, 67.6–74%) (Figure 4A), and the pooled specificity rate was 98.7% (95%CI, 98.2–99.0%) (Figure 5A). Pooled PLR was 31.5 (95% CI, 12.4–79.7) (Figure S1), whereas pooled NLR was 0.31 (95% CI, 0.24–0.40) (Figure S2). The DOR ranged from 55.0 to 351.3 (mean, 139.1) (Figure S3). An sROC curve was plotted to display sensitivity against 1—specificity for each individual study. The area under the curve (AUC) derived from the sROC curve was 0.93 and Q* was 0.8684 (Figure 6A).

#### 3.3.2. Meta-Analysis of the Group with Moderate Resistance Limited to 4–128 mg/L

Fourteen studies showed data for determining the accuracy of the detection of ML-AzmR *N. gonorrhoeae* isolates (MICs was 4–128 mg/L) based on the mutation C2611T. The sensitivity rates in these studies ranged from 40.0% to 100.0%. The pooled sensitivity rate of the molecular assays was determined to be 91.9% (95% CI, 88.9–94.2%) (Figure 4B), and the pooled specificity rate was 95.9% (95%CI, 95.1–96.6%) (Figure 5B). Pooled PLR was 12.1 (95% CI, 5.9–24.8) (Figure S4), whereas pooled NLR was 0.14 (95% CI, 0.01–0.23) (Figure S5). The DOR ranged from 41.9 to 364.1 (mean, 123.6) (Figure S6). An sROC curve was plotted to display sensitivity against 1—specificity from each individual study. The AUC derived from the sROC curve was 0.96 and the Q* was 0.9097 (Figure 6B).

#### 3.3.3. Analysis of the Causes of Heterogeneity in the Included Studies

##### Meta-Regression Analysis of the Group with Moderate Resistance Limited to 2–128 mg/L

The Spearman correlation coefficient (0.40, *p* = 0.08) indicated that there was no threshold effect. Further meta-regression analysis based on continuous variables, such as the detection method, isolates sampling (a random sample or not), location, and sample size indicated, that these factors were not the sources of heterogeneity (Table 4).

##### Meta-Regression Analysis of the Group with Moderate Resistance Limited to 4–128 mg/L

The Spearman correlation coefficient (−0.282, *p* = 0.329) indicated that there was no threshold effect. Further meta-regression analysis based on continuous variables such as the detection method, isolates sampling (as a random sample or not), location, and sample size indicated that these factors were not the sources of heterogeneity (Table 5).

### 3.4. Publication Bias

The results of the Deeks funnel plot asymmetry test showed that the *p*-value was 0.82 in the 2–128 mg/L group, and 0.76 in the 4–128 mg/L group, indicating that there was no publication bias in either group (Figures S7 and S8).

## 4. Discussion

To the best of our knowledge, this is the first published article focusing on the relationship between the mutation of C2611T in the 23S rRNA gene and moderate-level azithromycin resistance. In previous studies, the azithromycin MICs for *N. gonorrhoeae* were detected by the agar dilution and E-test methods, both methods having obvious disadvantages [4]. The agar dilution method is problematic in operation, and its results are affected by many factors, such as the composition of the agar medium, the pH value, and the culture parameters. Therefore, although the MICs estimated by different laboratories are comparable, there may be some random error in the values due to technical nuances that may affect the clinical interpretation. As far as the E-test method is concerned, the cost is very high. Due to the patent protection of the manufacturer, it requires the use of expensive experimental materials that are not available in some areas. The gold standard MIC-based agar dilution method and the E-test method are both based on subjective, visual readouts and are, therefore, limited to relatively low throughput. On the other hand, the molecular detection (the PCR and WGS methods) of mutant C2611T can be used as an alternative method to identify moderate-level azithromycin-resistant strains. However, it has not yet developed into a commercial diagnostic kit for clinical application. In this review, we systematically evaluated the accuracy of molecular tests for identifying moderate-level of azithromycin resistance to verify the association between the 23S rRNA mutation C2611T and moderate-level azithromycin resistance.

A total of twenty studies were included in the meta-analysis. The premise that the lower limit of the ML-AzmR *N. gonorrhoeae* isolates’ MIC is 2 mg/L or 4 mg/L is still controversial, we divided the meta-analysis into two groups (moderate-level drug resistance breakpoint was 2–128 mg/L and 4–128 mg/L) to extract the article data. In the 2–128 mg/L group, the meta-analysis of the mutation of C2611T for the diagnosis of middle-level azithromycin resistance showed that the pooled sensitivity was 71.9%, the pooled specificity was 98.7%, and the missed diagnosis rate was 28.1%. In the 4–128 mg/L group, the pooled sensitivity was 91.9%, the pooled specificity was 95.9%, and the missed diagnosis rate was lower than that of the 2–128 mg/L group. We also combined sensitivity and specificity rates to create the sROC curve. The AUC of the 2–128 mg/L group was 0.93, and the AUC of the 4–128 mg/L group was 0.96, indicating that the accuracy of the mutation of C2611T for the diagnosis of middle-level azithromycin resistance was 96% in the group of 4–128 mg/L, which was higher than that of the 2–128 mg/L group. On the basis of the above data, molecular detection of the C2611T mutation has high accuracy in the diagnosis of ML-AzmR *N. gonorrhoeae*, especially when the MIC range is 4–128 mg/L.

This study has many advantages. Methodologically, this is the first analysis to explore the relationship between the C2611T mutation and moderate-level azithromycin resistance. Our results confirm that the C2611T mutation is an important factor leading to the moderate level of azithromycin resistance of *Neisseria gonorrhoeae*. Previous studies [35] proved that there is a relationship between the A2059G mutation and a high level of azithromycin resistance. Molecular methods can be used in the detection of azithromycin resistance in *N. gonorrhoeae* by combining two molecular detection methods. Thus, with the development of molecular detection kits based on these two mutations, the 23S rRNA point mutation of azithromycin-resistant *N. gonorrhoeae* will become a clinically and routinely detected resistance phenotype such as PPNG and TRNG. In terms of clinical significance, the highly accurate molecular detection of clinical isolates, low-level azithromycin-resistant, and moderate- and high-level azithromycin-resistant bacteria can be identified in the early clinical stage, improving efficiency and precision in the treatment of patients. For low-level, drug-resistant, and azithromycin-sensitive strains, a single 2 g dose of azithromycin is effective. For moderate- and high-level drug-resistant strains, other drugs, such as third-generation cephalosporins, need to be used [36,37,38].

There are limitations to this study. The meta-analysis shows that there is a high degree of heterogeneity among the included studies. The Spearman correlation analysis suggests that there is no threshold effect. Meta-regression analysis shows that the detection method, isolates sampling, location, and sample size are not the sources of heterogeneity. A possible explanation for the heterogeneity is that the sample size of some studies was less than 100. However, the sample size after meta-regression analysis was not the source of heterogeneity. More high-quality studies with larger sample sizes may be needed in the future.

## 5. Conclusions

Molecular detection methods can quickly detect the specific gene mutation of clinical isolates. Through the efficient detection of resistant gene mutations, patients can be given the appropriate concentration of antibiotics, which can effectively inhibit the emergence of *N. gonorrhoeae* antibiotic resistance. This meta-analysis shows that the molecular diagnostic accuracy of the 2611 mutation of 23S rRNA is high, especially from 4–128 mg/L. Molecular detection methods have promising potential for use as a diagnostic kit for the rapid identification of ML-AzmR isolates in the clinic. More high-quality studies with larger samples are needed to confirm this finding.

## Figures and Tables

**Table 1:** Summary of different variables for the meta-analysis of the diagnostic test. ML-AzmR, moderate-level azithromycin-resistant.

Azithromycin Susceptibility	With Mutants at Position 2611	Without Mutants at Position 2611
ML-AzmR isolates	true positive	false negative
Non ML-AzmR isolates	false positive	true negative

**Table 2:** The data and characteristics of 20 studies whose moderate-level drug resistance breakpoint was 2–128 mg/L. WGS, whole-genome sequencing.

Study Number	Year, First Author	Location	Isolate Collection Period	Technique	Diagnostic Test Results of Molecular Assays			
					TP	FP	FN	TN
1	Stephanie 2010	U.K.	2001–2007	PCR	22	1	4	30
2	Vanessa 2014	Canada	2010–2013	PCR	17	0	0	2
3	Demczuk 2015	Canada	1989–2013	WGS	2	0	1	2
4	Ella 2015	Australia	2012	PCR	3	3	0	64
5	Belkacem 2016	France	2013–2014	PCR	3	0	2	67
6	Jacobsson 2016	Europe	2009–2014	WGS	72	0	1	4
7	Demczuk 2016	Canada	1997–2014	WGS	140	2	69	35
8	Johnson 2017	USA	2009–2010	WGS	24	0	0	2
9	Wind 2017	The Netherlands	2008–2015	PCR	62	0	7	73
10	Zhang 2017	Shenzhen, China	2011–2015	PCR	10	0	19	79
11	Harris 2018	Europe	2013	WGS	10	0	1	1043
12	Lee 2018	New Zealand	2014–2015	WGS	2	0	0	396
13	Ryan 2018	Ireland	2014–2016	WGS	5	1	3	34
14	Wan 2018	Nanjing, China	2013–2014	PCR	22	29	11	62
15	Whiley 2018	Australia	2017	PCR	24	4	4	85
16	Liu 2019	Taiwan	2001–2018	PCR	1	1	1	49
17	Thomas 2019	USA	2014–2016	WGS	58	8	38	545
18	Gernert 2020	USA	2017	WGS	63	1	57	289
19	Hadad 2020	Europe	2013	WGS	8	0	5	950
20	Peterson 2020	Canada	2009–2019	PCR	31	3	15	78

**Table 3:** The data and characteristics of 14 studies whose moderate-level drug resistance breakpoint was 4–128 mg/L.

Study Number	Year, First Author	Location	Isolate Collection Period	Technique	Diagnostic Test Results of Molecular Assays			
					TP	FP	FN	TN
1	Vanessa 2014	Canada	2010–2013	PCR	15	2	0	2
2	Demczuk 2015	Canada	1989–2013	WGS	1	1	1	2
3	Belkacem 2016	France	2013–2014	PCR	2	1	0	69
4	Jacobsson 2016	Europe	2009–2014	WGS	72	0	0	5
5	Demczuk 2016	Canada	1997–2014	WGS	129	13	6	98
6	Johnson 2017	USA	2009–2010	WGS	21	3	0	2
7	Harris 2018	Europe	2013	WGS	9	1	0	1044
8	Lee 2018	New Zealand	2014–2015	WGS	2	0	0	396
9	Ryan 2018	Ireland	2014–2016	WGS	5	1	2	35
10	Wan 2018	Nanjing, China	2013–2014	PCR	15	36	5	68
11	Whiley 2018	Australia	2017	PCR	9	19	0	89
12	Thomas 2019	USA	2014–2016	WGS	41	32	6	570
13	Gernert 2020	USA	2017	WGS	61	3	14	332
14	Peterson 2020	Canada	2009–2019	PCR	25	9	2	91

**Table 4:** Meta-regression analysis of the detection method, isolates sampling, location, and sample size of the group 2–128 mg/L. RDOR, relative diagnostic odds ratio.

Variables	Coefficient	*p*-Value	RDOR	95% CI
detection method	0.967	0.3102	2.63	(0.37; 18.83)
isolates sampling	0.003	0.9984	1.00	(0.06; 16.86)
location	0.802	0.4384	2.23	(0.26; 19.23)
sample size	−0.416	0.7281	0.66	(0.05; 8.17)

**Table 5:** Meta-regression analysis of the detection method, isolates sampling, location, and sample size of the group 2–128 mg/L.

Variables	Coefficient	*p*-Value	RDOR	95% CI
detection method	1.705	0.2457	5.50	(0.24; 127.00)
isolates sampling	1.565	0.2457	4.78	(0.07; 266.26)
location	0.375	0.8096	1.46	(0.05; 46.95)
sample size	−0.089	0.9611	0.91	(0.02; 53.97)

## Data Availability

Data is contained within the article or Supplementary Materials.

## Supplementary Materials

The following are available online at https://www.mdpi.com/article/10.3390/antibiotics10091027/s1, Figure S1: Forest plots for the combined positive LR from included studies with moderate resistance limited to 2–128 mg/L. LR, likelihood ratio.; Figure S2: Forest plots for the combined negative LR from included studies with moderate resistance limited to 2–128 mg/L. Figure S3: Forest plots for the combined diagnostic OR from included studies with moderate resistance limited to 2–128 mg/L. OR, odd ratio. Figure S4: Forest plots for the combined positive LR from included studies with moderate resistance limited to 4–128 mg/L. Figure S5: Forest plots for the combined negative LR from included studies with moderate resistance limited to 4–128 mg/L. Figure S6: Forest plots for the combined diagnostic OR from included studies with moderate resistance limited to 4–128 mg/L. Figure S7: Deeks’ funnel plot asymmetry test indicating the risk of publication bias of the group 2–128 mg/L. ESS, effective sample size. Figure S8: Deeks’ funnel plot asymmetry test indicating the risk of publication bias of the group 4–128 mg/L.
