# Supplementary material for: The Accuracy of Molecular Detection Targeting the Mutation C2611T for Detecting Moderate-Level Azithromycin Resistance in Neisseria gonorrhoeae: A Systematic Review and Meta-Analysis
Source: Antibiotics (Basel). 2021 Aug 24;10(9):1027. doi: 10.3390/antibiotics10091027 (PMC8471969; doi:10.3390/antibiotics10091027)
Supplement: Supplementary file 1 [file antibiotics-10-01027-s001.zip › antibiotics-1309728-supplementary.pdf]

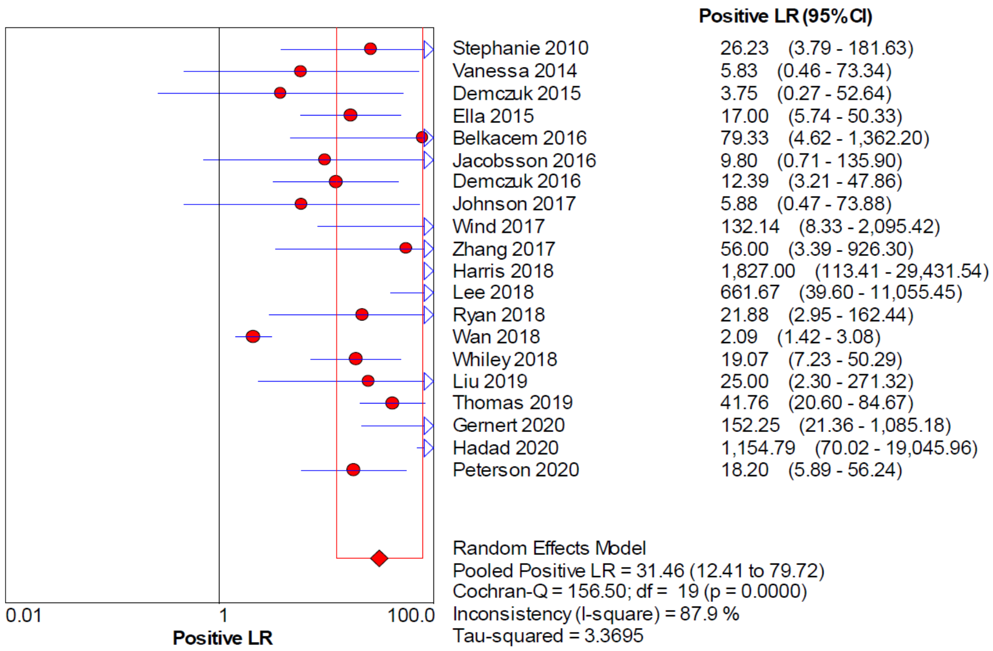

Figure S1. Forest plots for the combined positive LR from included studies with moderate resistance limited to 2-128mg/L. LR, likelihood ratio.

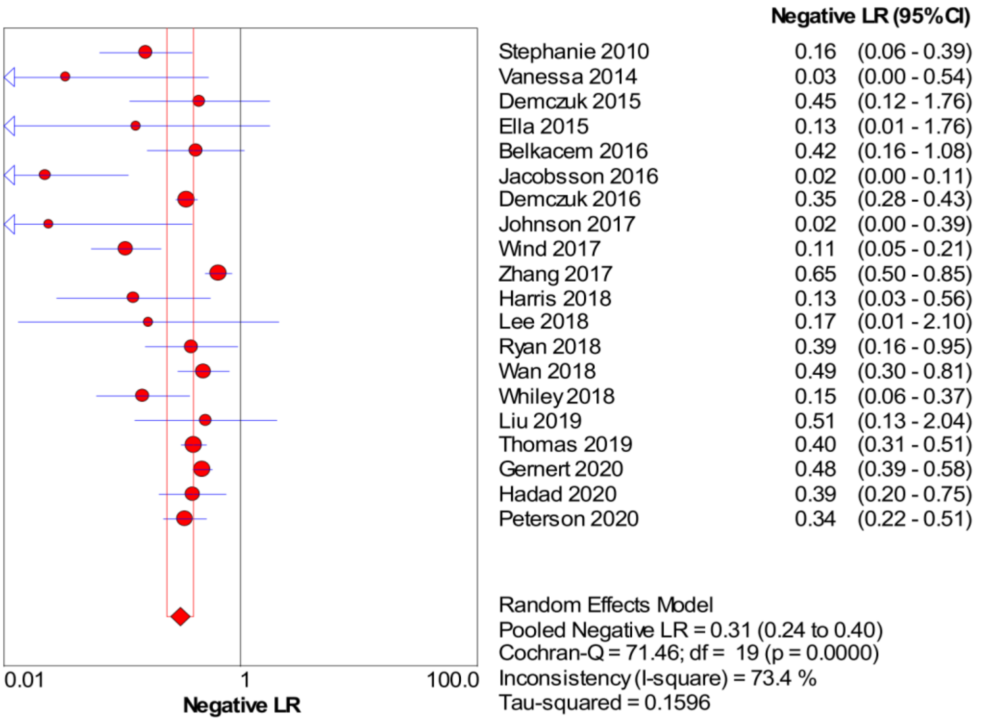

Figure S2. Forest plots for the combined negative LR from included studies with moderate resistance limited to 2-128mg/L.

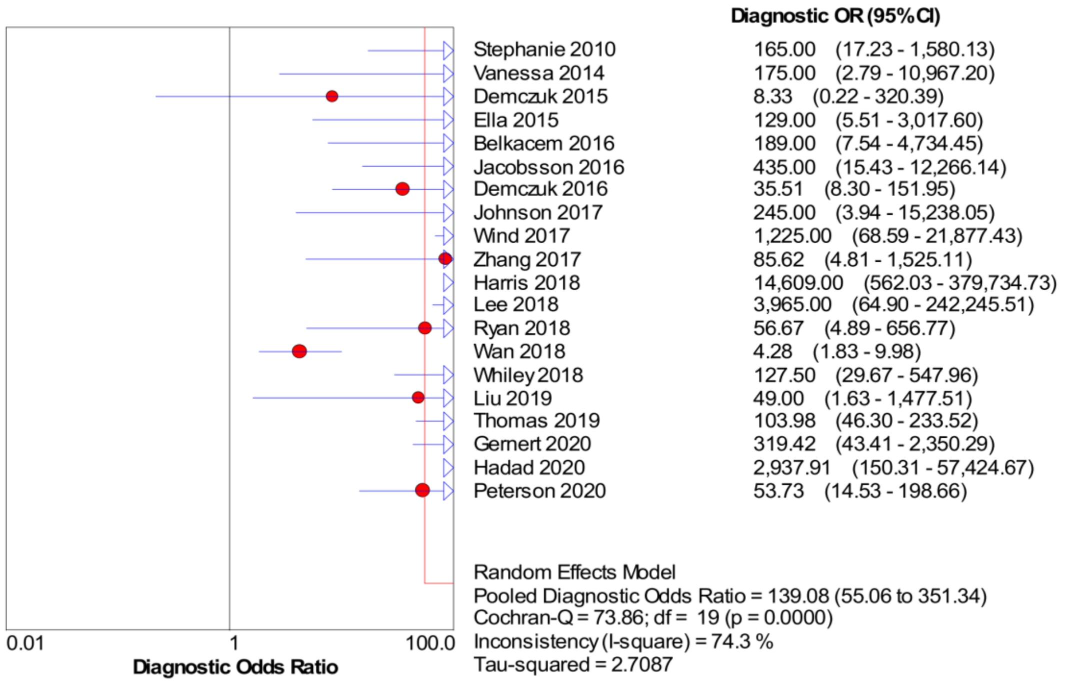

Figure S3. Forest plots for the combined diagnostic OR from included studies with moderate resistance limited to 2-128mg/L. OR, odd ratio.

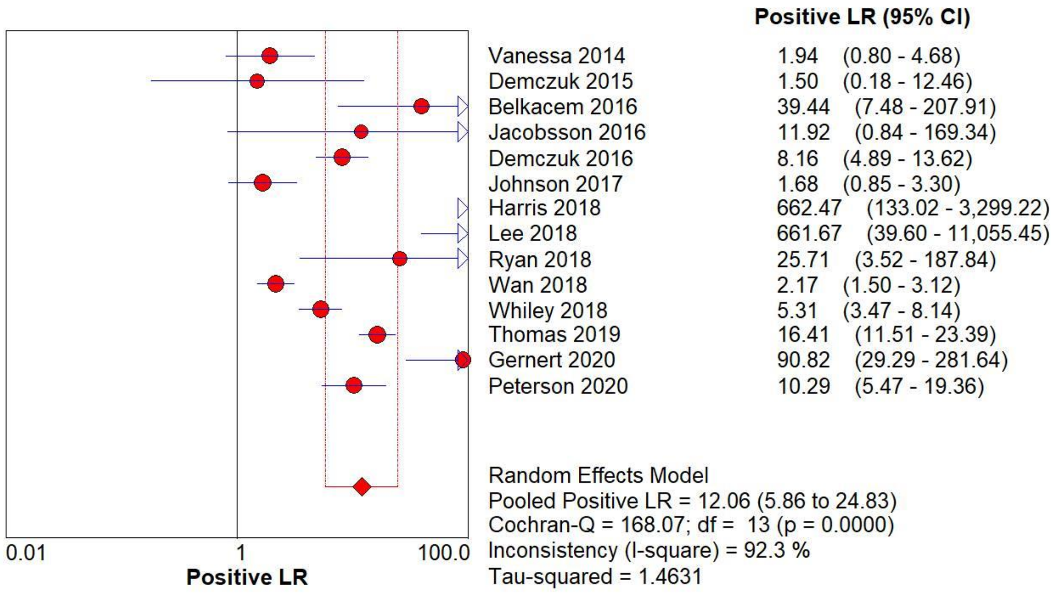

Figure S4. Forest plots for the combined positive LR from included studies with moderate resistance limited to 4-128mg/L.

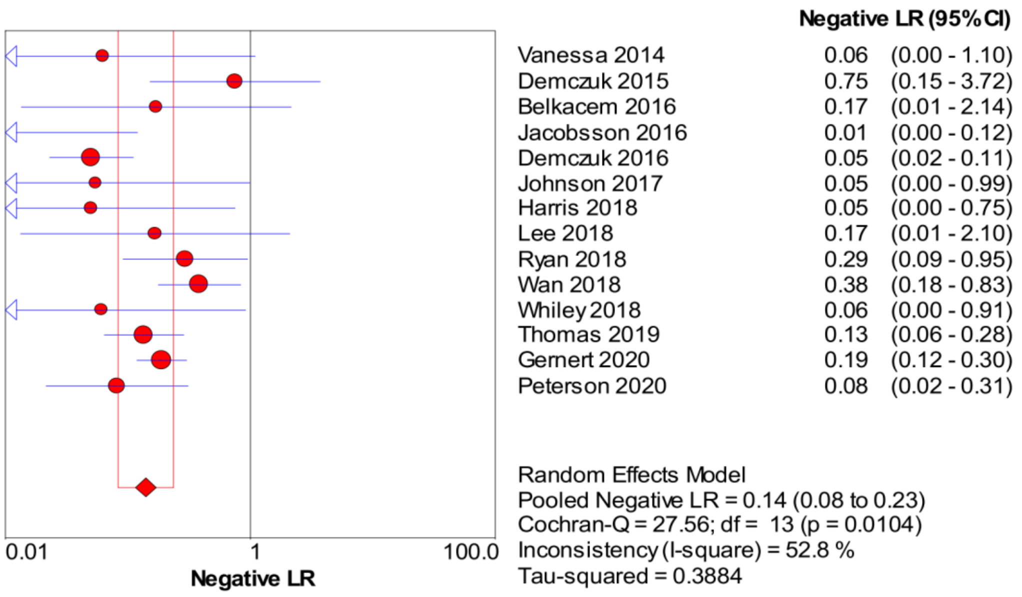

Figure S5. Forest plots for the combined negative LR from included studies with moderate resistance limited to 4-128mg/L.

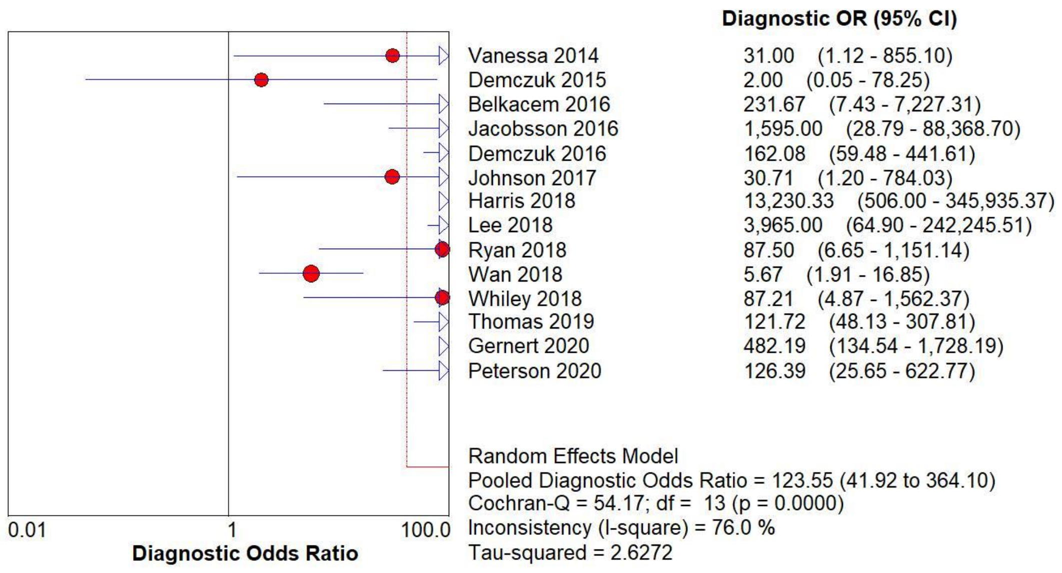

Figure S6. Forest plots for the combined diagnostic OR from included studies with moderate resistance limited to 4-128mg/L.

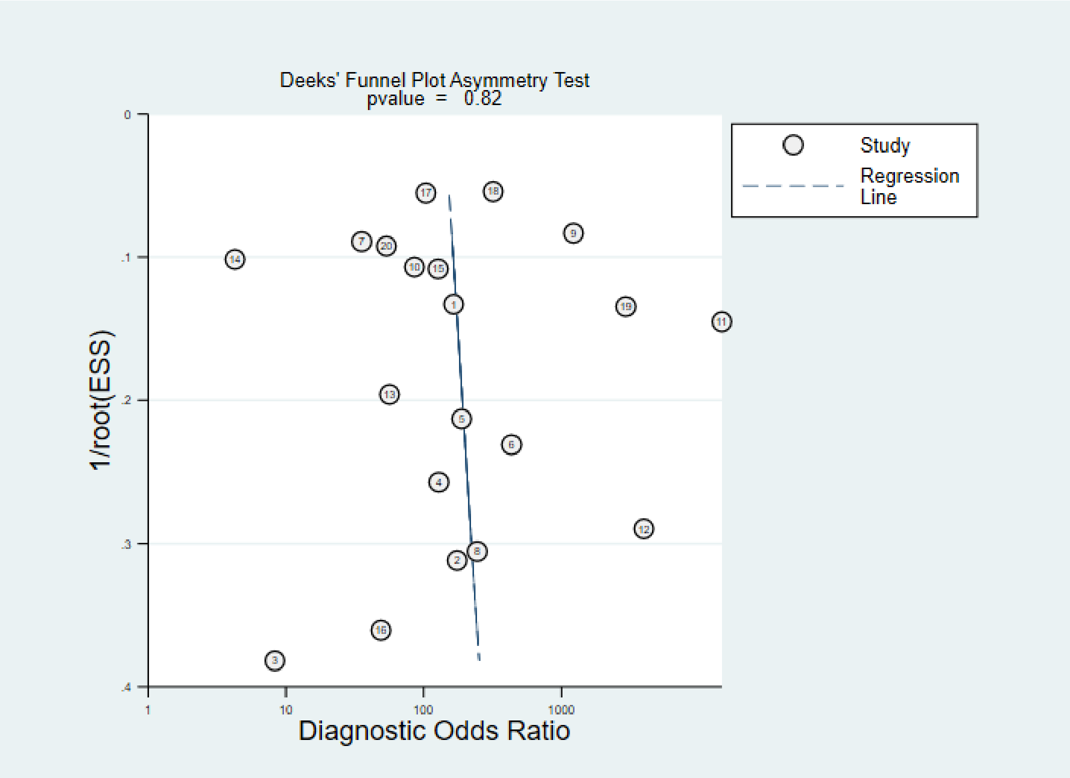

Figure S7. Deeks' funnel plot asymmetry test indicating the risk of publication bias of the group 2-128mg/L. ESS, effective sample size.

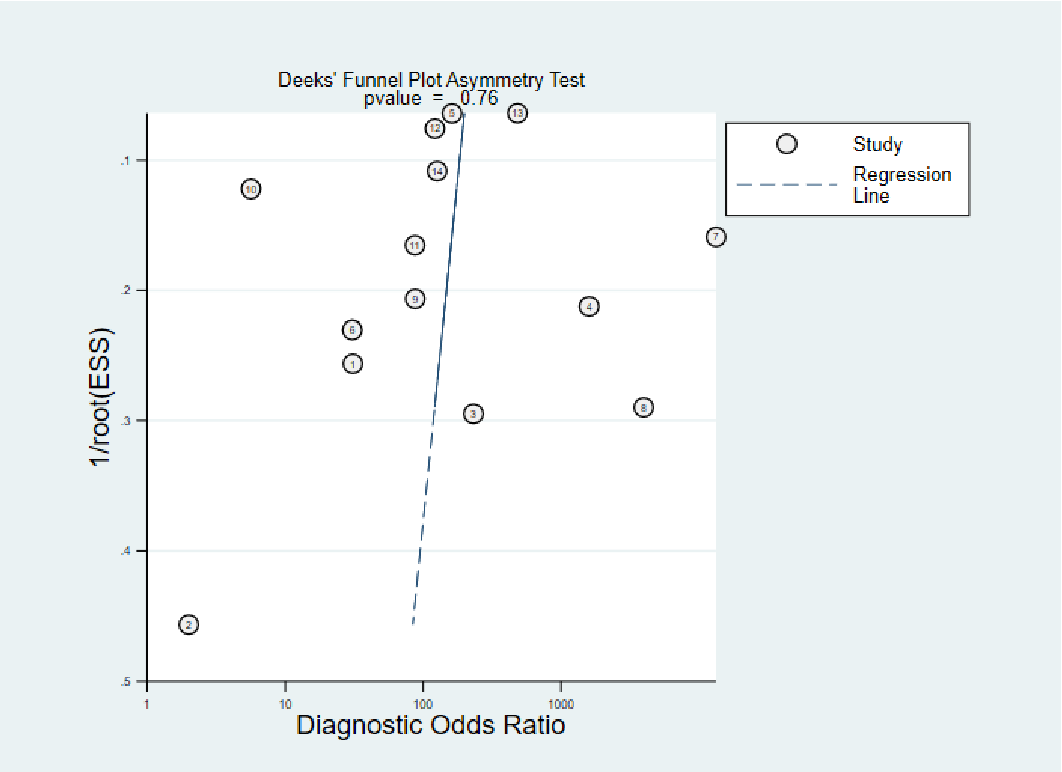

Figure S8. Deeks' funnel plot asymmetry test indicating the risk of publication bias of the group 4-128mg/L.
